# Population Exposure to Ambient PM_2.5_ at the Subdistrict Level in China

**DOI:** 10.3390/ijerph15122683

**Published:** 2018-11-28

**Authors:** Ying Long, Jianghao Wang, Kang Wu, Junjie Zhang

**Affiliations:** 1 School of Architecture and Hang Lung Center for Real Estate, Tsinghua University, Beijing 100084, China; 2 State Key Laboratory of Resources and Environmental Information System, Institute of Geographic Sciences and Natural Resources Research, Chinese Academy of Sciences, Beijing 100101, China; wangjh@lreis.ac.cn; 3 Beijing Key Laboratory of Megaregions Sustainable Development Modelling and School of Urban Economics and Public Administration, Capital University of Economics and Business, Beijing 100070, China; 4 Environmental Research Center, Duke Kunshan University, Kunshan 215316, China; junjie.zhang@duke.edu

**Keywords:** PM_2.5_, population exposure, MODIS (Moderate Resolution Imaging Spectroradiometer) AOD, China

## Abstract

Fine-particulate pollution is a major public health concern in China. Accurate assessment of the population exposed to PM_2.5_ requires high-resolution pollution and population information. This paper assesses China’s potential population exposure to PM_2.5_, maps its spatiotemporal variability, and simulates the effects of the recent air pollution control policy. We relate satellite-based Aerosol Optical Depth (AOD) retrievals to ground-based PM_2.5_ observations. We employ block cokriging (BCK) to improve the spatial interpolation of PM_2.5_ distribution. We use the subdistrict level population data to estimate and map the potential population exposure to PM_2.5_ pollution in China at the subdistrict level, the smallest administrative unit with public demographic information. During 8 April 2013 and 7 April 2014, China’s population-weighted annual average PM_2.5_ concentration was nearly 7 times the annual average level suggested by the World Health Organization (WHO). About 1322 million people, or 98.6% of the total population, were exposed to PM_2.5_ at levels above WHO’s daily guideline for longer than half a year. If China can achieve its Action Plan on Prevention and Control of Air Pollution targets by 2017, the population exposed to PM_2.5_ above China’s daily standard for longer than half a year will be reduced by 85%.

## 1. Introduction

Airborne particulate matter (PM) has become a major public health concern in China after three decades of rapid economic growth that heavily relies on fossil fuel consumption [1,2]. Epidemiological evidence consistently shows that PM pollution is associated with adverse health outcomes [3,4,5,6]. Fine particles with aerodynamic equivalent diameter less than 2.5 µm called Particulate Matter 2.5 (PM_2.5_) are particularly harmful [7]. According to the Global Burden of Disease Study 2010, ambient air pollution in China, largely driven by high levels of PM_2.5_, is the fourth leading risk factor for disability-adjusted life-years [8]. Therefore, many countries, including China, have identified PM_2.5_ as a key target in air quality management.

Although PM10 has been regulated for about two decades, China’s PM_2.5_ pollution did not draw national and international attention until a series of “airpocolypse” events in recent years. The frequent attacks of extreme air pollution episodes triggered the government to include fine particles in the revised air quality standards [9]. Pursuant to the new regulation, environmental protection agencies have systematically reported PM_2.5_ data since 2013. Using the first batch of ground-based observations, we assess the population exposure to PM_2.5_ with a fine spatiotemporal resolution at the national scale.

Accurate exposure assessment requires high-resolution PM_2.5_ data [10]. However, simple interpolation methods such as ordinary block kriging (BK) [11] that rely on sparsely located ground monitors are subject to large estimation errors. To address this challenge, satellite remote sensing has been used to improve the accuracy of spatial interpolation of PM_2.5_ concentrations [12,13,14]. The applicable approaches include exposure indicator variables, interpolation methods, dispersion models, and land use regression models [15]. Among these alternatives, the interpolation methods that incorporate ground-based measurements with satellite-retrieved Aerosol Optical Depth (AOD) are particularly promising [16,17]. Because AOD is intrinsically correlated with particulate matter concentrations, it provides secondary information about the spatial distribution of PM_2.5_ over the domain.

In this paper, we use AOD as auxiliary information to interpolate daily PM_2.5_ levels for each subdistrict. More specifically, we employ block cokriging (BCK) [11] to combine information from Moderate Resolution Imaging Spectroradiometer (MODIS) AOD retrievals [18,19,20] and ground-level PM_2.5_ measurements. One advantage of the BCK method is to estimate fine-scale spatial distribution of PM_2.5_ concentrations in high frequency (e.g., daily). Another advantage is to handle missing values in AOD retrievals caused by cloud or snow cover [20]. Without requiring AOD to cover the whole space exhaustively, the BCK method can still make full use of the spatial autocorrelation and cross correlation of both ground-level measurements and MODIS AOD retrievals. In order to assess model accuracy, we validate our model by means of the leave-one-out cross-validation (LOOCV) method.

The interpolated PM_2.5_ levels are used to calculate population exposure. Exposure is the contact between pollutant and the outer boundary of a human [21]. Because the information required for estimating actual exposure to PM_2.5_ for each subdistrict in China is overwhelming, we use simplified exposure indicators. We define exposure duration as the total number of days in a year exceeding certain daily PM_2.5_ standards, total exposure as the product of exposure duration and exposed population, and exposure intensity as the product of exposure duration and exposed population density. Although these three indicators are relatively rough estimates, they provide essential information about the population exposed to PM_2.5_ at a fine spatiotemporal resolution.

The dataset for this analysis is assembled from various sources. First, the ground-based observations of daily average PM_2.5_ concentrations were collected by web-scraping the website of the Ministry of Environmental Protection of China. The data cover the period of 8 April 2013 to 7 April 2014 for all 945 monitoring stations in 190 cities. Second, we obtained daily MODIS AOD Collection 5 from the Atmosphere Archive and Distribution System. We extracted AOD at the wavelength of 0.55 µm. Third, the subdistrict-level demographic information is from the 2010 National Population Census. Fourth, the boundaries data for 654 cities as 1:4,000,000 maps are obtained from China’s National Fundamental Geographical Information System.

To the best of our knowledge, we are the first to assess China’s potential population exposure to PM_2.5_ on a daily basis at the subdistrict level. In this paper, we use the term subdistrict to represent all types of township-level administrative units, which is the smallest administrative unit with public demographic information. Previous studies on China’s population exposure to PM_2.5_ only focus on one major metropolitan area, such as Beijing or Tianjin [22,23]. In comparison, our study covers the whole country at a fine spatial and temporal resolution, taking advantage of both ground-based PM_2.5_ observations and satellite remote sensing. In addition, by linking detailed PM_2.5_ pollution levels with fine-scale demographic data, it allows us to make more comprehensive and accurate assessment of the PM_2.5_ exposure.

## 2. Data

### 2.1. Ground-Based PM_2.5_ Measurements

China’s Ministry of Environmental Protection (MEP) and its local agencies maintain a nationwide network of air quality monitoring stations, which monitor concentrations of particulate and gaseous air pollutants at the ground level. Although fine particles are the most harmful components of particulate matters, PM_2.5_ has not been regulated until very recently due to technical and economic constraints. The 2012 Ambient Air Quality Standards and Technical Regulation on Ambient Air Quality Index required cities to report hourly concentrations of six criteria pollutants including PM_2.5_ in real time by January 2013. All provincial capitals and some municipalities in the developed regions are among the first group to disclose PM_2.5_ information. By the end of 2013, China had established 945 monitoring stations, increasing from 670 in 2012. We have collected daily average PM_2.5_ concentrations from 8 April 2013 and 7 April 2014 from all monitoring stations in 190 cities. The one year of in situ observations at daily level for each station are used as a key input for pollution exposure estimation.

**Figure 1:**
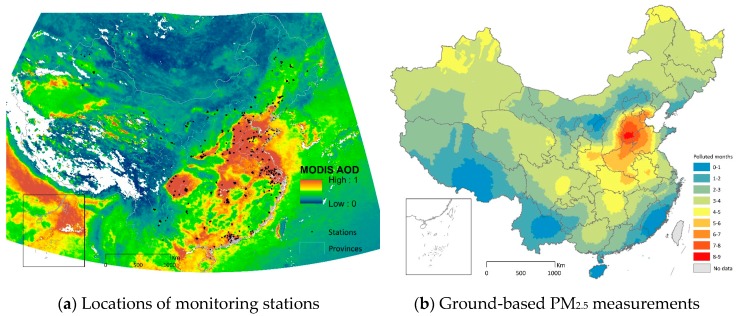
Panel (**a**): spatial distribution of monitoring stations in 190 Chinese cities and Moderate Resolution Imaging Spectroradiometer (MODIS) Aerosol Optical Depth (AOD) at 550 nm average from 8 April 2013 to 7 April 2014 as the background of this panel. By the end of 2013, 945 stations have disclosed PM_2.5_ concentrations to the public. Panel (**b**): the duration of PM_2.5_ above China’s daily standard spatially interpolated by ground-based observations only. We convert the number of pollution days to months by assuming 30 days in a month.

Ground-based monitoring stations are sparsely located. Figure 1a shows the locations of all monitoring sites. Figure 1b is the spatial interpolation of the duration of PM_2.5_ above China’s daily standard, which is obtained by applying the kriging method to the ground-based observations only. We notice that a similar approach has been used by the Chinese government to illustrate the spatial pattern of smog duration. However, the spatial resolution of the interpolated PM_2.5_, which relies on ground-based observations only, is very coarse.

### 2.2. MODIS AOD Retrievals

Satellite remote sensing has been used to estimate PM_2.5_ concentrations in the areas lacking ground-level measurements [12,13]. The MODIS sensor is one of the first passive satellite radiometers designed to systematically retrieve aerosol properties over both land and ocean on a daily basis [18]. NASA released global retrievals of MODIS AOD with the nadir resolution of 10 km. The uncertainty of the MODIS derived AOD is expected to be ±(0.05 + 15%) over the land [19]. In the MODIS AOD Collection 5 retrieval algorithm, three different channels—0.47, 0.66, and 2.12 µm—are primarily employed for over-land aerosol retrievals. These three wavelength channels are simultaneously inverted to finally report AOD values at the wavelength of 0.55 µm. More details about the retrievals of the MODIS AOD product are discussed in Remer et al. [18] and Levy et al. [20]. In this paper, we use the MODIS AOD retrievals to improve daily PM_2.5_ estimates for the whole country. We obtained the daily MODIS AOD Collection 5 from the Atmosphere Archive and Distribution System. These AOD retrievals were screened from cloud and bright surfaces. We extracted AOD at 0.55 µm as auxiliary information to spatially interpolate PM_2.5_ levels.

### 2.3. Population

The population density data for each subdistrict were obtained from the 6th National Population Census in 2010, which covers the People’s Republic of China but does not include Hong Kong, Macau, and Taiwan. China has three forms of township-level administrative units: subdistrict (*jiedao*), town (*zhen*), and township (*xiang*). Subdistrict is mainly in cities; its counterpart in suburbs and rural areas is town or township. Hereafter in this paper, we use the term subdistrict to represent all types of township-level administrative units in China. The subdistrict boundary data are obtained from the National Data Sharing Infrastructure of Earth System Science (www.geodata.cn). A subdistrict includes dozens of census units and it is the smallest population unit available to the public. As of 2010, China has about 40,000 subdistricts; average subdistrict population density is close to 1 thousand persons per km^2^. In the 2010 census, the population is divided into three segments: children (0–14), adults (15–64), and seniors (≥65), which we admit it is not consistent with commonly adopted age segmentations, e.g., 0–18, 18–64, and above 65. The age information allows us to assess exposure for the most susceptible members of population, such as children and seniors.

### 2.4. City Boundaries

We analyze and rank the population exposure to PM_2.5_ in each city by aggregating the subdistrict-level data to the city level. The city boundaries data, in the form of 1:4,000,000 maps, are obtained from the National Fundamental Geographical Information System of China. Our study covers 654 cities in China, in which 286 cities are at the prefecture level or above and the rest are county-level cities. It is worth noting that a Chinese city proper contains both rural and urban land uses.

## 3. Method

### 3.1. Interpolating PM_2.5_ Concentrations

One challenge to map high-resolution PM_2.5_ concentrations is the sparsely located monitoring stations. To address this challenge, satellite-retrieved AOD has been used to improve the accuracy of spatial interpolation of PM_2.5_ concentrations. In general, there are mainly two ways to estimate PM_2.5_ from ground monitoring stations with satellite-retrieved AOD. One approach uses AOD retrievals as a covariate to predict ground-level PM_2.5_ concentrations with general regression models [16]. The other approach uses AOD retrievals as auxiliary information in various types of kriging algorithms [17]. In this paper, we employ block cokriging (BCK) algorithms [11] to improve PM_2.5_ concentration interpolation by combining information from daily MODIS AOD retrievals and ground-level measurements.

Let *Z*_1_ designate PM_2.5_ ground observations and *Z*_2_ designate MODIS AOD retrievals. Since *Z*_1_ and *Z*_2_ are spatially and mutually correlated, the spatial variation of *Z*_2_ can be used to predict that of *Z*_1_. BCK estimates the average value of the variable *Z*_1_ over an area *v* with covariate *Z*_2_ as

(1) $${\hat{Z}}_{1}(v)=\sum_{i=1}^{n_{1}}\lambda_{1i}(v)Z_{1}(s_{1i})+\sum_{i=1}^{n_{2}}\lambda_{2i}(v)Z_{2}(s_{2i})$$

where *s*_1_ and *s*_2_ denote set of spatial coordinates, *n*_1_ and *n*_2_ are the number of observations for *Z*_1_ and *Z*_2_, respectively. The above estimator is unbiased if the BCK weights $\lambda_{1i}(v)$ and $\lambda_{2i}(v)$ satisfy the following constraints:

(2) $$\sum_{i=1}^{n_{1}}\lambda_{1i}(v)=1\;and\;\sum_{i=1}^{n_{2}}\lambda_{2i}(v)=0$$

The system of Equation (1) determining the weights in the BCK estimator is obtained by imposing two constraints that require the estimator to be unbiased and efficient [11,24]. Using the solution of this system, we can derive the estimates and standard errors for the BCK prediction of PM_2.5_ concentrations at the subdistrict level.

Our model is validated by the LOOCV method. It works as follows. The model predicts an annual time series of daily PM_2.5_ concentrations for each station using the observations from its neighboring stations. BCK uses both ground-level observations and MODIS AOD retrievals; BK (block kriging) uses ground-level observations only. The predicted PM_2.5_ concentration is compared with the actual PM_2.5_ observation to derive the cross-validation statistics. Model accuracy is assessed by using the Pearson’s correlation coefficient (COR) and the root-mean-square error (RMSE):

(3) $$\mathrm{RMSE}=\sqrt{\frac{1}{n}\sum_{i=1}^{n}{\left({\hat{Z}}_{1}(v_{i})-Z_{1}(v_{i})\right)}^{2}}$$

In this form, *n* is the number of observations, ${\hat{Z}}_{1}(v_{i})$ is the cross-validation estimate, and $Z_{1}(v_{i})$ is the observed PM_2.5_ concentration at subdistrict *i*. In model comparisons, the preferred model is the one associated with higher COR and smaller RMSE.

### 3.2. Potential Population Exposure

Our exposure measurement is formed by the interaction of the number of pollution days and the exposed population. We define a pollution day as its 24-h average of PM_2.5_ concentrations exceeding China’s or WHO’s air quality standard. Let *v* index subdistrict, *t* index day, *c* designate daily average PM_2.5_ concentration, *C* designate PM_2.5_ standard, and *p* designate the size of population or population segment. The total exposure *E* for subdistrict *v* over a period of time *τ* = *t*_2_ − *t*_1_ is given by

(4) $$E_{v}=\sum_{t=t_{1}}^{t_{2}}p_{v}1\left(c_{vt}\geq C\right)$$

where 1() is an indicator function of whether PM_2.5_ concentration of a particular day is above the standard. Total exposure (person days) measures the aggregated public health risk of PM_2.5_ in any given area or period. However, this indicator is not appropriate for the mapping purpose because land areas vary hugely among subdistricts and cities; the large areas will be visually over-represented on the map. Therefore, we use exposure intensity *EI* to map PM_2.5_ exposure for each subdistrict:

(5) $${EI}_{v}=D_{v}^{\tau }\frac{p_{v}}{a_{v}}$$

In this form, *a_v_* is the land area of subdistrict *v* and $D_{V}^{\tau }=\sum_{t=t_{1}}^{t_{2}}1\left(c_{vt}\geq C\right)$ is the exposure duration (number of pollution days) in period *τ*.

## 4. Results

By applying BCK to ground-level PM_2.5_ measurements and MODIS AOD retrievals, we obtain 365 maps for interpolated PM_2.5_ concentrations at the subdistrict level. The daily mean time series of PM_2.5_ concentration over China is shown in Supplementary Materials, Figure S3. For comparison, we also apply BK, which uses the ground data only, to generate daily PM_2.5_ concentrations. We validate our model using the LOOCV method. Model accuracy, as well as model comparison, is assessed using Pearson’s correlation coefficient (COR) and root-mean-square error (RMSE). The time series of RMSE are illustrated in Supplementary Materials, Figure S4. The validation results show that the yearly mean COR for BK and BCK is 0.832 and 0.864 respectively; the yearly mean RMSE for BK and BCK is 21.5 µg/m^3^ and 19.6 µg/m^3^ respectively. Since the preferred model is associated with higher COR and smaller RMSE, both statistics indicate that BCK fits the data very well; it also improves the accuracy of PM_2.5_ estimation compared with BK.

We find that Chinese people were exposed to serious PM_2.5_ pollution between 8 April 2013 and 7 April 2014. The population-weighted annual average PM_2.5_ concentration reaches 68.3 µg/m^3^. It is nearly 7 times the annual average level of 10 µg/m^3^ recommended by WHO. Using China’s daily standard of 75 µg/m^3^, an average Chinese person experienced 113 pollution days in the past year. If we use the WHO’s daily guideline of 25 µg/m^3^, the exposure duration increases to 257 days in that year.

**Figure 2:**
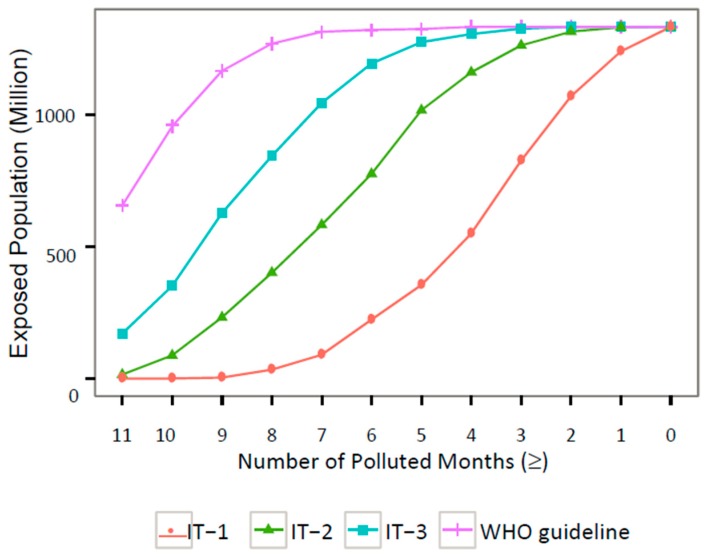
Exposure duration and exposed population under various ambient air quality standards. The x-axis represents exposure duration that measures the cumulative time exceeding certain PM_2.5_ standard. The y-axis represents exposed population. Each standard is represented by one colored curve. A dot (*x*, *y*) on a curve s means that y million people are exposed to PM_2.5_ above the standard s for over x months.

WHO suggests three interim target (IT) levels for the daily average PM_2.5_ concentration [25]: 75 µg/m^3^ (IT-1), 50 µg/m^3^ (IT-2), and 37.5 µg/m^3^ (IT-3). Population exposures under various standards are illustrated in Figure 2. It shows that the amount of exposed population increases dramatically as the applicable standard becomes more stringent. The simulation results demonstrate that 223 million people were exposed to PM_2.5_ above China’s ambient PM_2.5_ standard (or IT-1) for longer than half a year. The exposed population increases to 776 or 1195 million if IT-2 or IT-3 level is used. Using the WHO guideline of 25 µg/m^3^, 1322 million people, or 98.6% of the Chinese population in 2010, were exposed to PM_2.5_ above WHO’s daily standard for over half a year. Additional exposure measurements are reported in the Supplementary Materials, Table S1.

### 4.1. Spatiotemporal Variation

**Figure 3:**
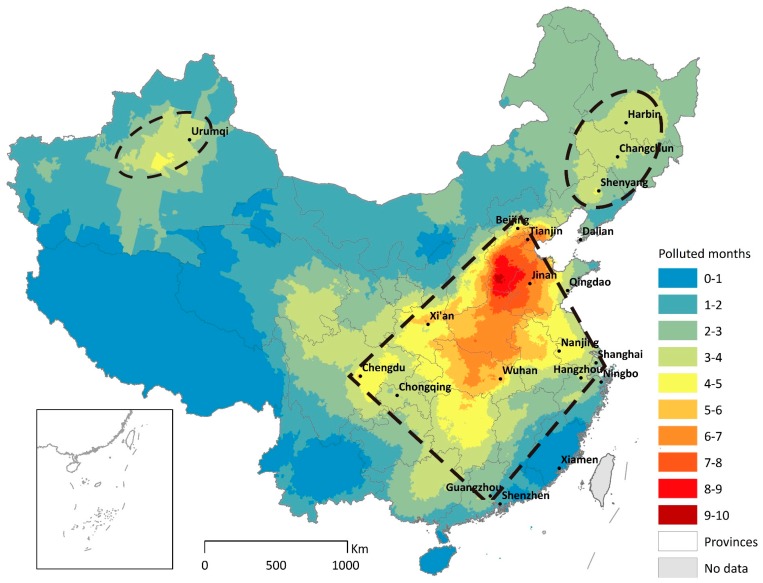
China’s three major PM_2.5_ pollution hotspots. The color bar measures the total number of days with daily average PM_2.5_ concentration exceeding China’s daily standard from 8 April 2013 to 7 April 2014. The number of pollution days is converted to months by a factor of 30. Taiwan, Hong Kong, and Macau are colored in grey due to the missing data. The spatial resolution is subdistrict.

We illustrate the spatial heterogeneity of PM_2.5_ by aggregating exposure duration to the yearly level in Figure 3. The most pronounced PM_2.5_ hotspot is a diamond-shaped large area that sprawls across eastern and central China. The area is anchored by four major metropolitan areas: Beijing in the north, Shanghai in the east, Guangzhou in the south, and Chengdu in the west. The pollution center is formed due to a combination of factors, such as fossil fuel combustion, industrial processes, and natural conditions. We estimate exposure duration for 654 cities (Supplementary Materials, Figure S1). The result shows that northern cities are worse than southern cities, inland cities are worse than coastal cities, and plain and basin cities are worse than plateau and hilly cities. In addition, we rank major cities and city regions by PM_2.5_ exposure and discuss the spatial pattern in the Supplementary Materials, Tables S2–S4.

**Figure 4:**
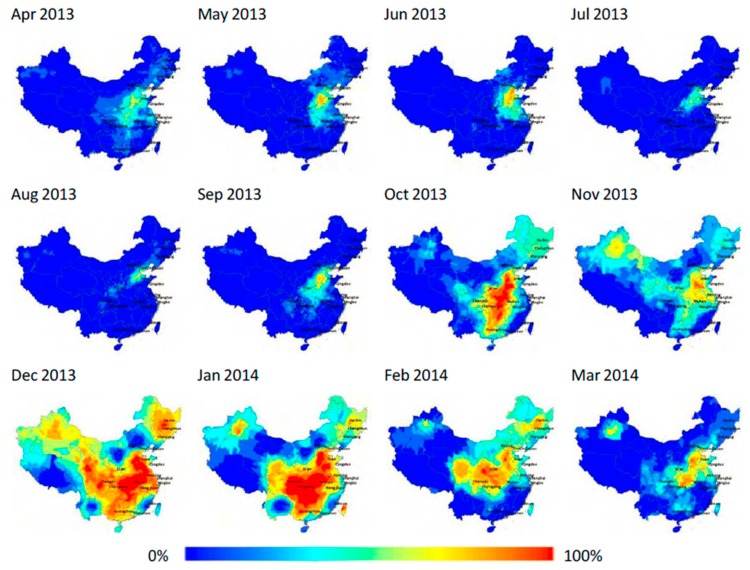
Spatiotemporal variation of PM_2.5_ exposure duration. The color ramp represents the percentage of days with PM_2.5_ concentration exceeding China’s daily standard for each month between 8 April 2013 and 7 April 2014. No ground observations are used for Taiwan, Hong Kong, and Macau. The spatial resolution is subdistrict.

We demonstrate the seasonality of PM_2.5_ by calculating the percentage of days within a month that exceeds China’s daily PM_2.5_ standard. Figure 4 illustrates the spatiotemporal variation of exposure duration. The seasonal fluctuation spreads and congregates in space due to a complex interplay of weather variability, diffusion conditions, and coal combustion [26]. Overall, PM_2.5_ pollution of winter half year (from October to March) is much more severe than that of summer half year (from April to September). The whole country is exposed to high levels of PM_2.5_ in December and January due to the influence of downdraft and coal-fired heating [27,28]. Pollution starts to abate during February and reaches the lowest level in August. In spring and summer, pollution is limited to a number of areas in the north due to energy consumption structure or heavy industries development and spring dust storms occasionally [29]. However, PM_2.5_ in North China still has a high level even between May and September. Pollution starts to increase again after August; it gradually expands from the north to the south until it covers most parts of China in December.

### 4.2. Population Segments

Studies reveal that children and seniors are more susceptible to the adverse health effects of PM_2.5_ pollution [30,31]. Therefore, we estimate the exposure intensity for different population segments including children (≤14) and seniors (≥65). We calculate exposure intensity based on the exposure duration and population density in each subdistrict. Because PM_2.5_ and population density are positively correlated, nearly all densely populated subdistricts are among the worst polluted regions. Therefore, exposure intensity is more spatially concentrated compared to population density.

**Figure 5:**
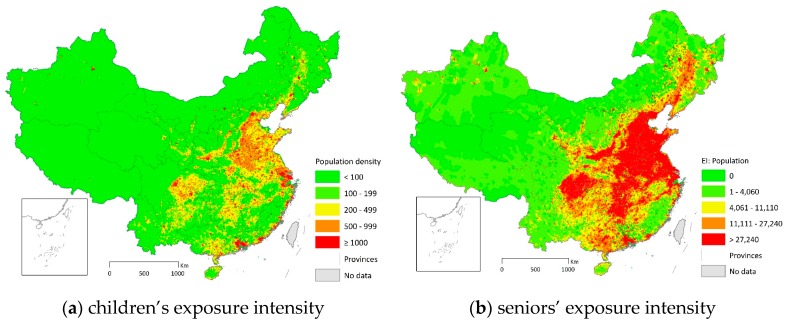
Exposure intensity (pollution days*persons per km^2^) for the susceptible subpopulation: (**a**) children (≤14), and (**b**) seniors (≥65). Exposure intensity is the product of exposure duration and exposed population density. Exposure duration is the total number of days in a year exceeding China’s daily PM_2.5_ standard. The spatial resolution is subdistrict.

The spatial distribution of each population segment is slightly different. The correlation matrix in the Supplementary Materials, Table S5, shows that children on average have higher risk of PM_2.5_ exposure than the average population. This is perhaps due to the high birth rate in the more populated and then more polluted areas. The condition for the senior subpopulation, however, is better than the average and young population. This might be explained by the fact that seniors tend to stay in places with better environment. Another possible explanation is that adults between 15 and 64 are likely to migrate to population centers for better job opportunities, which turn out to also be pollution hotspots. The exposure intensity for children and seniors is mapped in Figure 5. The figure reveals different spatial patterns for children and seniors. Nevertheless, the exposure intensity for the susceptible subpopulation (children and seniors combined) has a similar pattern with that of the total population (Supplementary Materials, Figure S2).

### 4.3. Policy Simulation

We simulate the effects of the most recent air pollution control policy: the Action Plan on Prevention and Control of Air Pollution (hereinafter referred to as the Action Plan). The Action Plan is arguably the toughest regulation on PM pollution in Chinese history. It stipulates differentiated targets for all provinces (Supplementary Materials, Table S6). The three major metropolitan areas—Beijing-Tianjin-Hebei, Yangtze River Delta, and Pearl River Delta—are required to reduce their annual average PM_2.5_ concentrations by 25%, 20%, and 15% by 2017, respectively. The second-tier provinces are required to reduce annual average PM10 concentrations. The remaining provinces have no quantified targets but are required to make continuous improvements.

**Figure 6:**
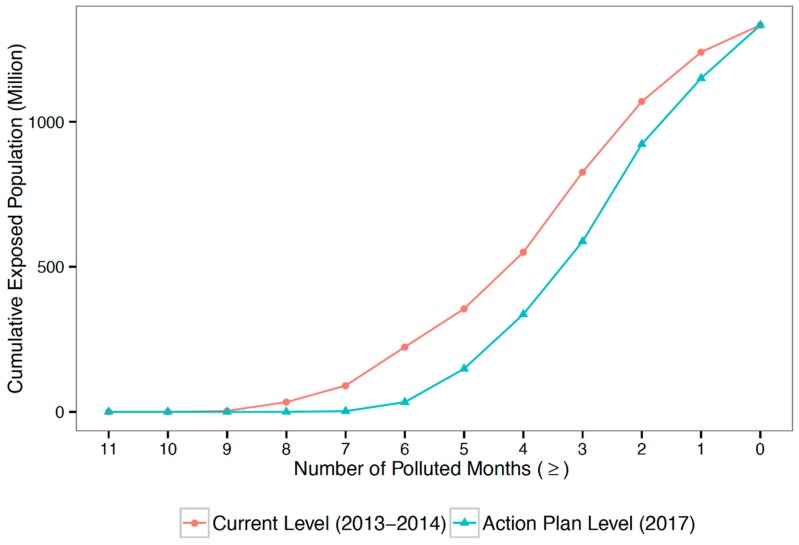
Simulation results for the effects of the Action Plan. Both curves use China’s daily PM_2.5_ standard. The interpretation is the same as in Figure 2.

The simulation result in Figure 6 demonstrates that the population exposure to PM_2.5_ will be reduced significantly if China can successfully achieve its targets in the Action Plan. Based on China’s daily standard, the population exposed to PM_2.5_ above the standard for over half a year will be 33 million people; the population-weighted average exposure duration will be 86 days. Compared with 223 million people and 113 days of exposure during 2013–2014, the PM_2.5_ exposure risk by 2017 will be significantly lower.

In particular, the population-weighted annual average PM_2.5_ concentration will be reduced from 68.3 µg/m^3^ (2013–2014) to 58.4 µg/m^3^ (2017). The PM-mortality effect estimated in the Huai River study suggests that a 30 µg/m^3^ increase in PM_2.5_ is associated with 3 years of shortened life expectancy [32,33]. Based on this estimate, the Action Plan, through reducing PM_2.5_ concentration by about 10 µg/m3, can avoid approximately a 1-year loss of life expectancy for an average Chinese person.

Our simulation assumes that the population density in 2017 is the same as that in 2010. However, migration and urbanization can shift population densities across space over the period 2010–2017. In order to assess the sensitivity of our results to the assumption of population density, we conduct a counterfactual analysis by using 2010 population density and 2000 population density (Supplementary Materials, Table S7). The result shows that the shifting population density during 2000–2010 does not significantly affect our estimated population exposure, suggesting that our results are robust to the assumption of population density.

## 5. Discussion

PM_2.5_ data are critical for epidemiological, environmental, urban planning, and economic studies. However, their applications in China have been hampered by the limited availability of pollution data, especially those with fine resolution. This study contributes to China’s air pollution studies by systematically documenting, synthesizing, and sharing fine-resolution PM_2.5_ data. The output of this paper—maps, data, and results—is disclosed to the public at the website of the Beijing City Lab (BCL), in order to provide better information for citizens, researchers, policy makers, and firms to address the challenge of fine-particulate matter pollution.

It is worth noting that BCK is only one of the spatial statistical techniques that can take advantage of both ground- and satellite-based observations. More studies recently have used land-use regression (LUR) models [15,16] or geographically weighted regression (GWR) models [14] to estimate local PM_2.5_ concentrations with spatially predicting variables. Both LUR and GWR models require the covariates to have spatially exhaustive coverage. Satellite-retrieved AOD, as one of the most crucial covariates in regression models, however, commonly has large missing values due to cloud or snow cover [20]. Therefore, it is a challenge to estimate fine-scale PM_2.5_ concentrations at a high frequency (e.g., daily) with large missing values in AOD by using regression models. BCK has a distinct advantage that it does not need AOD to exhaustively cover space. Therefore, it can make full use of cross correlation with MODIS AOD to optimally estimate values at each sub-district per day.

The fine-scale PM_2.5_ data generated by this paper can be used in many other studies. One direction is to link with time-stamped and geocoded health data like hospital admission and discharge data [34]. This enables us to study the PM_2.5_—health relationship at the national scale with a fine resolution, which can facilitate holistic policy analysis. Another direction is to analyze the driving factors of PM_2.5_ pollution, such as the factors related to urban form [35]. Our estimates of PM_2.5_ concentrations with high resolution are essential to understand intra-urban heterogeneities, which can inform planners to design better urban form to improve environmental quality.

Please note that our study has several potential caveats. The first concern is about the data quality of the ground-based observations. A recent study finds suggestive evidence that a large number of Chinese cities are suspected to manipulate PM10 data around the cutoff for “blue-sky days” during 2001–2010 [36]. Since 2010, the credibility of air pollution data has improved significantly thanks to the direct online reporting system that largely prevents data falsification. We compared the PM_2.5_ data reported by the MEP and the U.S. Embassy in Beijing. The pollution distributions have no significant difference. The results are illustrated in Figure S5.

Second, our measurement of population exposure to PM_2.5_ does not take into account avoidance behavior such as canceling outdoor activities, wearing face masks, and installing air purifiers. The behavioral adaptation will reduce actual pollution exposure and hence mitigate the negative health impacts of PM_2.5_. In addition, our analysis does not involve indoor air pollution, which is another major cause of particulate matter exposure [37]. An accurate assessment has to account for the duration of indoor and outdoor activities. Thus, our indicator of potential pollution exposure is likely to be the upper bound of the actual exposure.

Third, our estimates of pollution exposure are associated with large uncertainty in the locations where ground monitoring stations are sparse. Over 400 Chinese cities have no monitoring stations. Their PM_2.5_ concentrations are heavily determined by the MODIS AOD images. The best way to improve the estimation in these cities is to increase the number of monitoring stations, which calls for additional investment from the central and local governments.

## 6. Conclusions

In this paper, we assess China’s potential population exposure to PM_2.5_, map its spatiotemporal variability, and simulate the effects of the recent air pollution control policy. We find that about 1322 million people, or 98.6% of the total population, are exposed to PM_2.5_ above the WHO daily guideline for longer than half a year during 2013–2014. The proposed Action Plan to tackle fine particles is costly, which requires a total investment of US$277.5 billion over the next 5 years [9]. However, the benefit of air quality improvement is also tremendous. If China can successfully achieve its targets in the Action Plan by 2017, it will reduce population-weighted annual average PM_2.5_ concentration by about 10 µg/m^3^, which might avoid an approximate 1-year loss of life expectancy for an average Chinese person based on the recent estimate of PM mortality [32,33]. With a population over 1.3 billion, the Action Plan can justify its hefty cost of fine-particulate pollution control. Therefore, the successful implementation of the Action Plan can significantly reduce PM_2.5_ exposure risk. However, the annual PM_2.5_ concentration by 2017, which is about 6 times the WHO guideline, is still at a high level harmful to public health.

## Supplementary Materials

The following are available online at http://www.mdpi.com/1660-4601/15/12/2683/s1, Table S1: Potential Exposure to PM_2.5_, Table S2: PM_2.5_ pollution in major city regions, Table S3: Top 20 best and worst Chinese cities in PM_2.5_ exposure duration, Table S4: Ranking of Chinese cities in potential population exposure to PM_2.5_ pollution, Table S5: Pearson correlation matrix for population density and exposure duration, Table S6: Provincial targets under the Air Pollution Prevention and Control Action Plan, Table S7: Exposure estimation using 2010 and 2000 population density; Figure S1: Exposure duration (polluted days) at the city level. Exposure duration is the total number of days in a year exceeding China’s current PM_2.5_ standard (Figure produced from ArcGIS 10.2), Figure S2: Exposure intensity (person days per km^2^) for (A) total population and (B) susceptible subpopulation (children and seniors). The spatial resolution is subdistrict (Figure produced from ArcGIS 10.2), Figure S3: The time series of daily mean PM_2.5_ concentration in China, Figure S4: The time series of daily RMSE of PM_2.5_ concentration estimation with BCK, Figure S5: The distributions of PM_2.5_ concentrations of the MEP and the U.S. Embassy in Beijing.
