# Supplementary material for: Population Exposure to Ambient PM2.5 at the Subdistrict Level in China
Source: Int J Environ Res Public Health. 2018 Nov 28;15(12):2683. doi: 10.3390/ijerph15122683 (PMC6313548; doi:10.3390/ijerph15122683)
Supplement: Supplementary file 1 [file ijerph-15-02683-s001.pdf]

Supplementary Materials

Urban Areas and City Regions

Since air pollution is primarily an urban challenge, we aggregate the subdistrict pollution level to the city level. Figure S1 in the supplementary appendix illustrates the spatial heterogeneity of $PM_{2.5}$ pollution across 654 cities. The pollution pattern shows that northern cities are worse than southern cities, inland cities are worse than coastal cities, and plain and basin cities are worse than plateau and hilly cities.

We use total exposure, a product of exposure duration and population, to rank cities in $PM_{2.5}$ pollution. We report the rank of the best and worst cities in terms of $PM_{2.5}$ pollution in Table S3 in the supplementary appendix.[1] We find that the worst 20 cities are mostly from central or southern part of Hebei province, which is clustered with the iron and steel industry that heavily relies on coal consumption. Beijing is one of the most polluted cities in terms of total exposure, indicating a huge amount of the population is exposed to $PM_{2.5}$ pollution. The other three megacities–Tianjin, Shanghai, and Chongqing–are all top the list too. Although some cities have a relatively shorter duration of exposure, the risk of pollution is still high because of the concentrated population. In contrast, Tibet, Yunnan, and Fujian have the cleanest cities in China in terms of $PM_{2.5}$.

Furthermore, we focus on $PM_{2.5}$ exposures in the thirteen major city regions that are identified by the Ministry of Environmental Protection of China as the key regions in air pollution control. Table S2 presents various exposure indicators. The results show that half of these city regions experienced over 100 pollution days in the past year. Only Fujian province has a relatively low annual average concentration of 37 $\mu g/m^3$, which is still slightly above the national standard of 35$\mu g/m^3$. The percentage of cities in these city regions that comply with China's $PM_{2.5}$ annual standard is negligible.

[1] The cities in the Beijing-Tianjin-Hebei (BTH) region , Henan, mid-western Shandong (except cities in the Shandong Peninsula), central Hubei, and central Shaanxi (eg, Xi'an) are ranked the top on the list of most polluted cities. The next tier of polluted cities with exposure between 111 and 158 days include the northeastern Yangtze River Delta (YRD, eg, Nanjing), Chengdu Plain (eg, Chengdu), east Hubei, and Hunan (eg, Wuhan and Changsha). Residents in the major urbanization city-regions like BTH, YRD, Chengdu-Chongqing, and the middle reaches of Yangtze River are exposed to $PM_{2.5}$ concentrations above the standard for more than 100 days a year. The exception is that the Pearl River Delta (PRD), east Fujian, Shandong Peninsula, and Liaodong Peninsula have relatively less $PM_{2.5}$ pollution.

Table S1. Potential Exposure to $PM_{2.5}$.

Exposure Duration (Month)	Cumulative Exposure	
	Area (10,000 km^2)	Population (Million People)
≥1	745	1241
≥2	470	1070
≥3	243	827
≥4	110	550
≥5	61	355
≥6	35	223
≥7	12	90
≥8	4	34
≥9	0.4	3

Notes: Exposure duration is the total number of days above China's ambient $PM_{2.5}$ standard, which is converted to the equivalent number of months by a factor of 30.

Table S2. $PM_{2.5}$ pollution in major city regions.

City Region	Exposure Duration (day)	Annual Average Concentration (μg/m³)	Rate of Compliance (%)
Beijing-Tianjin-Hebei	219	107	0
Yangtze River Delta	99	64	0
Pearl River Delta	53	44	4.5
South-Central Liaoning	80	56	0
Shandong	146	80	0
Wuhan metropolitan area	161	87	0
Changsha-Zhuzhou-Xiangtan	127	71	0
Chengdu-Chongqing	113	66	0
Fujian	17	37	43.5
North Central Shanxi	128	70	0
Central Shaanxi	132	79	0
Gansu and Ningxia	69	58	0
Northern Xinjiang	89	60	0

Notes: Exposure duration is the average number of days above China's ambient $PM_{2.5}$ standard for the cities in the same city region. Annual average concentration is the simple arithmetic mean of $PM_{2.5}$ concentration between April 2013 and April 2014. Rate of compliance measures the percentage of cities in the city-region that comply with China's annual $PM_{2.5}$ standard.

Table S3. Top 20 best and worst Chinese cities in $PM_{2.5}$ exposure duration.

Top 20 Worst Cities			Top 20 Best Cities		
**City**	**Population (million)**	**Exposure Duration (day)**	**City**	**Population (million)**	**Exposure Duration (day)**
Xingtai	0.7	279	Lhasa	0.3	2
Shahe	0.5	260	Shigatse	0.1	2
Nangong	0.5	255	Anning	0.3	6
Handan	1.4	253	Yuxi	0.5	7
Linqing	0.7	253	Xiamen	3.5	8
Shijiazhuang	2.6	252	Nanan	1.4	8
Jizhou	0.4	252	Quanzhou	1.5	9
Gaocheng	0.8	250	Jinjiang	1.9	10
Jinzhou	0.5	248	Zhangping	0.2	10
Anyang	0.6	246	Zhangzhou	0.5	10
Xinji	0.6	245	Kunming	3.5	11
Wuan	0.8	244	Shishi	0.6	11
Hengshui	0.5	241	Longhai	1	11
Dezhou	0.6	240	Chuxiong	0.6	11
Xinle	0.5	239	Longyan	0.7	11
Luquan	0.4	238	Putian	1.9	12
Shenzhou	0.6	238	Ruili	0.2	14
Liaocheng	1.1	238	Yongan	0.3	14
Anguo	0.4	238	Fuqing	1.2	15
Yucheng	0.5	238	Fuzhou	2.9	15

Notes: The population is for the whole county if a city is a county-level city. For other cities with higher administrative rank, the population is for the city proper.

Table S4. Ranking of Chinese cities in potential population exposure to $PM_{2.5}$ pollution.

City	Population (million)	Exposure Duration (day)	Total Exposure (million people*day)	Annual Average Concentration (μg/m³)	Area (km²)
Beijing	18.9	161	3048	84	12,163
Tianjin	10.4	204	2130	94	7158
Shanghai	22.4	88	1964	60	5476
Wuhan	9.7	158	1535	85	8583
Chengdu	7.4	150	1114	81	2171
Chongqing	11.4	97	1104	61	15,385
Xi'an	6.5	162	1056	91	3569
Nanjing	7.2	138	991	76	4736
Jinan	4.1	213	873	98	3070
Zhengzhou	4.1	201	832	96	1015
Guangzhou	11.1	65	723	50	3412
Shenyang	6.3	108	676	64	3471
Harbin	5.8	115	668	69	7016
Tangshan	3.2	205	653	96	3253
Shijiazhuang	2.6	252	645	136	379
Hangzhou	6.3	98	619	66	3344
Zibo	3.1	188	589	91	2984
Suzhou	5.3	109	581	68	4606
Foshan	7.4	75	551	52	3798
Xuzhou	3.1	148	451	80	3038

Notes: The area is for the administrative boundary of each city, rather than the urban built-up area.

Table S5. Pearson correlation matrix for population density and exposure duration.

Variable	Population Density	Population Density (0–14)	Population Density (≥65)	Exposure Duration
Population density	1	0.942	0.949	0.116
Population density (0–14)		1	0.829	0.120
Population density (≥65)			1	0.109
Exposure duration				1

Notes: all correlation coefficients are significant at the 1% level. N=39,007.

Table S6. Provincial targets under the Air Pollution Prevention and Control Action Plan.

Regions	Target
**Group A: annual average $PM_{2.5}$ concentrations**	
Beijing, Tianjin, Hebei	-25%
Shanxi, Shanghai, Jiangsu, Shandong, Zhejiang	-20%
Guangdong (Pearl River Delta, PRD), Chongqing	-15%
Inner Mongolia	-10%
**Group B: annual average PM_{10} concentrations**	
Henan, Shaanxi, Qinghai, Xinjiang	-15%
Hubei, Gansu	-12%
Liaoning, Jilin, Anhui, Hunan, Guangdong (non-PRD), Sichuan, Ningxia	-10%
Heilongjiang, Fujian, Jiangxi, Guangxi, Guizhou	-5%
**Group C: requirement of continuous improvement**	
Hainan, Yunnan, Tibet	

Notes: The data are assembled from the implementation details of the Action Plan: the Responsibility Agreement on Air Pollution Control Targets signed between the Ministry of Environmental Protection and provinces. In the simulation, for the provinces with PM_{10} targets only, we assume that $PM_{2.5}$ is reduced proportionally with PM_{10}. In addition, daily concentration reduction is proportional to annual reduction. For the provinces without quantified target, we assume its $PM_{2.5}$ concentration stays at the same level.

Table S7. Exposure estimation using 2010 and 2000 population density.

Exposure Duration (month)	Cumulative Population Exposed (million)	
	2010 Population Density	2000 Population Density
≥0	1334	1334
≥1	1241	1244
≥2	1070	1084
≥3	827	848
≥4	550	560
≥5	355	357
≥6	223	226
≥7	90	90
≥8	34	33
≥9	3	3
≥10	0	0
≥11	0	0

Notes: Exposure duration measures the cumulative time exceeding China's current $PM_{2.5}$ standard. The second column used the census data in 2010. The third column is a counterfactual analysis that uses total population in 2010 and population density in 2000.

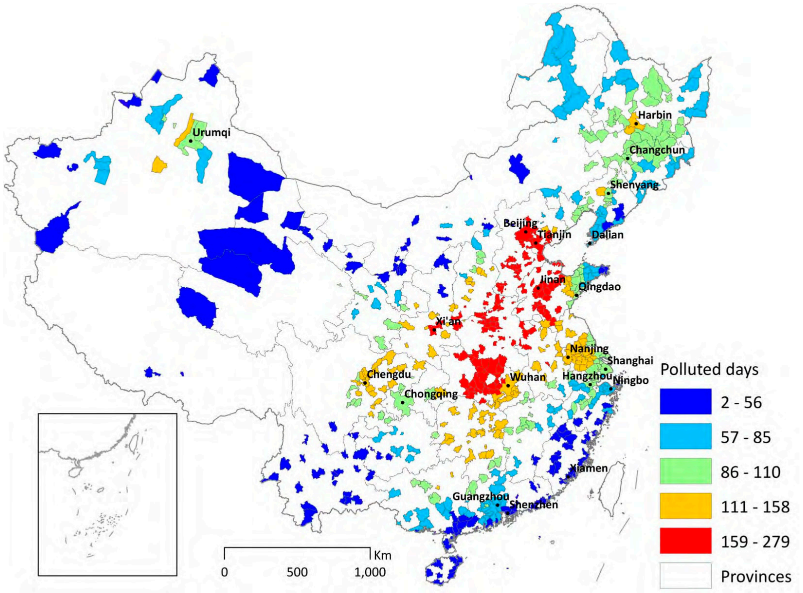

Figure S1. Exposure duration (polluted days) at the city level. Exposure duration is the total number of days in a year exceeding China's current $PM_{2.5}$ standard (Figure produced from ArcGIS 10.2).

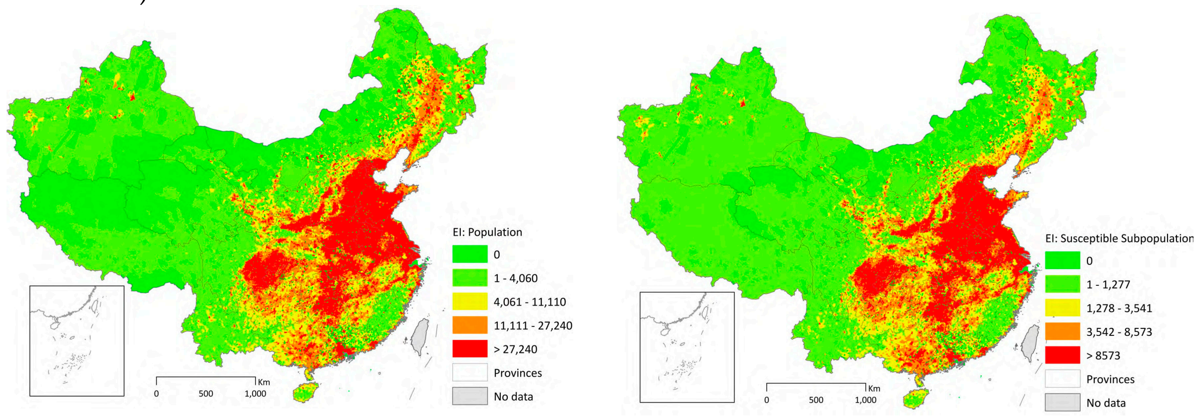

(A) Total population (B) Susceptible subpopulation

Figure S2. Exposure intensity (person days per km^2) for (**A**) total population and (**B**) susceptible subpopulation (children and seniors). The spatial resolution is subdistrict (Figure produced from ArcGIS 10.2).

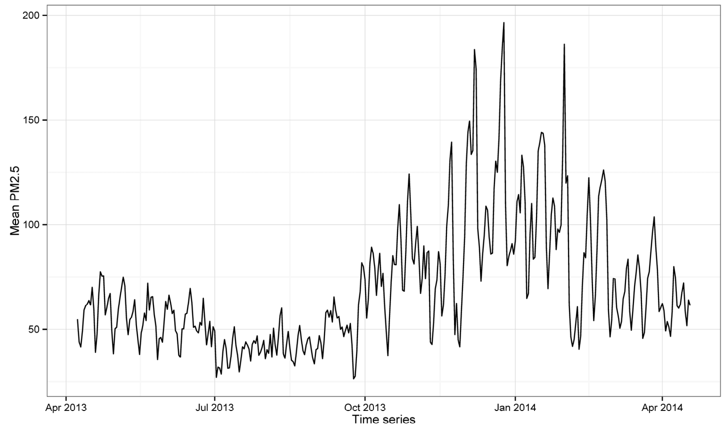

Figure S3. The time series of daily mean PM2.5 concentration in China.

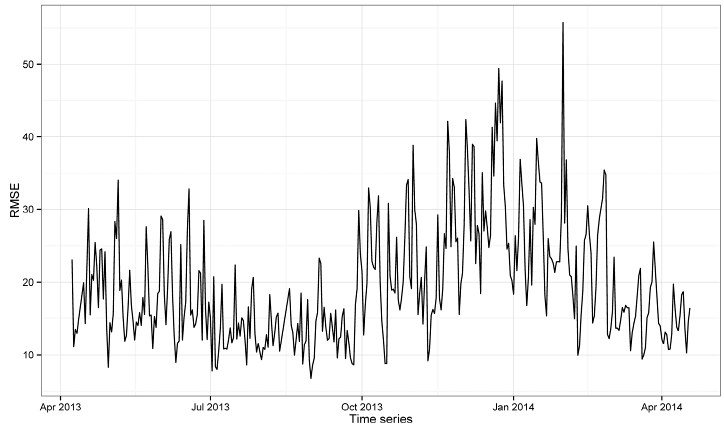

Figure S4. The time series of daily RMSE of PM2.5 concentration estimation with BCK. RMSE: root-mean-square error; BCK: block cokriging.

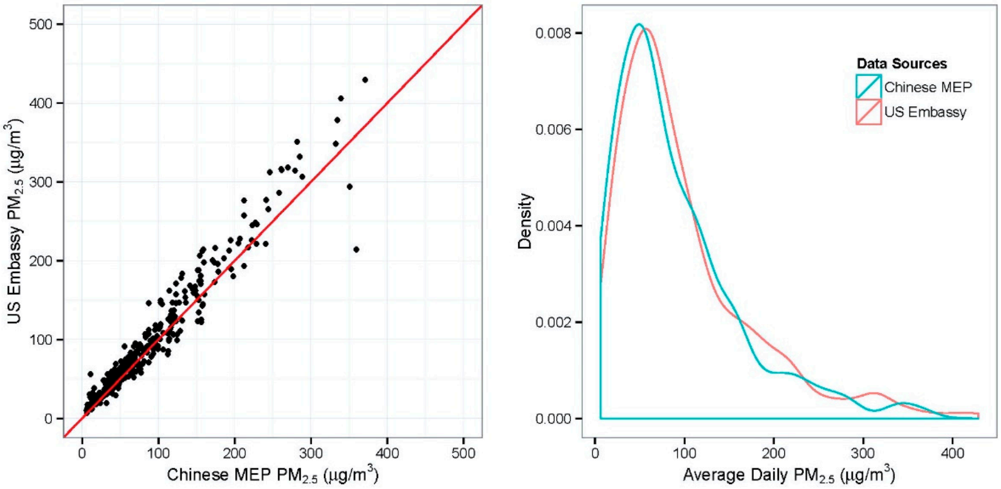

Figure S5. The distributions of PM2.5 concentrations of the MEP and the U.S. Embassy in Beijing. MEP: China's Ministry of Environmental Protection.
